# Terrestrial adaptation of green algae *Klebsormidium* and *Zygnema* (Charophyta) involves diversity in photosynthetic traits but not in CO_2_ acquisition

**DOI:** 10.1007/s00425-017-2741-5

**Published:** 2017-07-18

**Authors:** Mattia Pierangelini, David Ryšánek, Ingeborg Lang, Wolfram Adlassnig, Andreas Holzinger

**Affiliations:** 1 Department of Botany, Functional Plant Biology, University of Innsbruck, 6020 Innsbruck, Austria; 2 Department of Botany, Faculty of Science, Charles University in Prague, Benátská 2, 12801 Prague 2, Czech Republic; 3 Laboratory of Environmental Microbiology, Institute of Microbiology of the CAS, v. v. i., Průmyslová 595, 252 42 Vestec, Czech Republic; 4 Faculty of Life Sciences, Core Facility Cell Imaging and Ultrastructure Research, University of Vienna, Althanstrasse 14, 1090 Vienna, Austria

**Keywords:** Desiccation, Green algae, Light, Photosynthesis

## Abstract

**The basal streptophyte** ***Klebsormidium*** **and the advanced** ***Zygnema*** **show adaptation to terrestrialization. Differences are found in photoprotection and resistance to short-term light changes, but not in CO**_**2**_ **acquisition.**

Streptophyte green algae colonized land about 450–500 million years ago giving origin to terrestrial plants. We aim to understand how their physiological adaptations are linked to the ecological conditions (light, water and CO_2_) characterizing modern terrestrial habitats. A new *Klebsormidium* isolate from a strongly acidic environment of a former copper mine (Schwarzwand, Austria) is investigated, in comparison to *Klebsormidium* cf. *flaccidum* and *Zygnema* sp. We show that these genera possess different photosynthetic traits and water requirements. Particularly, the *Klebsormidium* species displayed a higher photoprotection capacity, concluded from non-photochemical quenching (NPQ) and higher tolerance to high light intensity than *Zygnema*. However, *Klebsormidium* suffered from photoinhibition when the light intensity in the environment increased rapidly, indicating that NPQ is involved in photoprotection against strong and stable irradiance. *Klebsormidium* was also highly resistant to cellular water loss (dehydration) under low light. On the other hand, exposure to relatively high light intensity during dehydration caused a harmful over-reduction of the electron transport chain, leading to PSII damages and impairing the ability to recover after rehydration. Thus, we suggest that dehydration is a selective force shaping the adaptation of this species towards low light. Contrary to the photosynthetic characteristics, the inorganic carbon (C_*i*_) acquisition was equivalent between *Klebsormidium* and *Zygnema*. Despite their different habitats and restriction to hydro-terrestrial environment, the three organisms showed similar use of CO_2_ and HCO_3_^−^ as source of C_i_ for photosynthesis, pointing out a similar adaptation of their CO_2_-concentrating mechanisms to terrestrial life.

**Electronic supplementary material:**

The online version of this article (doi:10.1007/s00425-017-2741-5) contains supplementary material, which is available to authorized users.

## Introduction

Streptophyte green algae started land colonization about 450–500 million years ago (MYA) and this was an important step for the evolution of terrestrial plants (Becker and Marin 2009; Becker 2013). With the transition to land, ancestors of these organisms had to face new environmental conditions, including exposure to higher solar irradiance compared to the water environment, lower water accessibility and higher pCO_2_ than in the extant atmosphere (Becker and Marin 2009; Alboresi et al. 2010; Raven and Colmer 2016). Among streptophytes, *Klebsormidium* (Klebsormidiophyceae) and *Zygnema* (Zygnematophyceae) first appeared ~500–700 MYA (Leliaert et al. 2011; Becker 2013) and they are classified as belonging to the basal and advanced groups of the streptophyte lineage, respectively (de Vries et al. 2016). The *Klebsormidium* genome has revealed that this organism acquired many genes specific for a plant terrestrial life (Hori et al. 2014; de Vries et al. 2017). Currently, *Zygnema*, as belonging to the order of Zygnematales, is among the closest algal relatives of land plants (Timme et al. 2012).

The pioneering behaviour of streptophyte green algae during land colonization is still present in modern habitats where they are abundant in freshwater, hydro-terrestrial habitats (*Zygnema*; Holzinger and Pichrtová 2016) and biological soil crusts (*Klebsormidium*; Holzinger and Karsten 2013; Karsten and Holzinger 2014) worldwide, and where they contribute to important ecological roles as primary production, carbon and nitrogen biogeochemical cycles, and soil stabilization (Elbert et al. 2012). Occurrence in these environments expose cells to various and extreme environmental conditions including long exposure to high light intensities and cellular water loss i.e., dehydration (Holzinger and Pichrtová 2016). Under such conditions, photosynthetic resistance against intense light involves the presence of photoprotective mechanisms e.g., energy dissipation as heat (non-photochemical quenching, NPQ; Alboresi et al. 2008; Gerotto et al. 2011; Goss and Lepetit 2015) and/or activation of a nonradiative electron recombination route to reduce ROS production (Ohad et al. 2010; Treves et al. 2016). *Klebsormidium* and *Zygnema* have been shown to have different NPQ kinetics that might confer a different sensitivity of their photosynthetic apparatus to high light environments (Gerotto and Morosinotto 2013).

As well as for light, not less important is photosynthetic resistance during dehydration (Heber 2008). In the case of *Klebsormidium*, transcriptomic analysis showed that dehydration caused up-regulation of genes involved in photosynthesis, showing that the photosynthetic apparatus is prone to acclimation under this condition (Holzinger et al. 2014). However, in nature, dehydration may occur rapidly, without giving time for the transcripts to be translated into proteins and to the cell, the chance to acclimate (Cruz de Carvalho et al. 2014). Moreover, due to the variability of light climate (e.g., daily light changes), frequently dehydration occurs under elevated irradiances which might pose a danger for photosynthesis (Gray et al. 2007; Raanan et al. 2016a). Consequently, high desiccation tolerant species must possess constitutively expressed mechanisms to quickly protect the photosynthetic apparatus and allowing recovery when water is taken up again (Gray et al. 2007; Yamakawa et al. 2012; Bar-Eyal et al. 2015). It is known that *Klebsormidium* is capable to inactivate photosynthesis during dehydration in low light (40 µmol photons m^−2^ s^−1^) and recover after rehydration (Herburger and Holzinger 2015; Karsten et al. 2016). However, to better understand its physiological requirements and distribution in the environment, we need to understand the response of its photosynthetic apparatus to dehydration when exposed to different light intensities.

Upon land colonization, besides high light and dehydration tolerance, adaptations for resources acquisition such as inorganic carbon (C_i_) are essential to guarantee the species occurrence in a certain environment. C_i_ acquisition has been extensively studied in aquatic algae and many of them are known to possess CO_2_-concentrating mechanisms (CCMs) i.e., structural and functional components whose role is to furnish the cell with CO_2_ for photosynthesis (Giordano et al. 2005; Reinfelder 2011). Different CCMs are observed among different species, in relation to the form of C_i_ uptaken (CO_2_ vs HCO_3_^−^) and presence or absence of pyrenoids (Ratti et al. 2007; Brading et al. 2013; Stojkovic et al. 2013). In several streptophyte green algae, including *Klebsormidium* and *Zygnema,* functional CCMs can be inferred from the presence of pyrenoids (Meyer et al. 2008; Herburger et al. 2015; Mikhailyuk et al. 2015), an organelle which, although with some exceptions, is often associated with intracellular C_i_ accumulation and CCM (Smith and Griffiths 1996; Maberly et al. 2009; Villarreal and Renner 2012; Raven et al. 2017). In the case of *Zygnema*, active CCM are also indicated by low CO_2_ compensation point (Birmingham and Colman 1979). In relation to the preferred C_i_ forms, the evolutionary adaptation of these organisms to terrestrial conditions may have favoured their predilection for CO_2_ acquisition rather than HCO_3_^−^. Yet, different preferences for CO_2_ or HCO_3_^−^ may be expected in relation to single species adaptation to a particular habitat (Lachmann et al. 2016). For instance, in species such as *Zygnema* with higher restriction to moist environments (Herburger and Holzinger 2015; Lajos et al. 2016), where both CO_2_ and HCO_3_^−^ are present, lower dependence on CO_2_ can be expected. Contrary, for species with higher adaptation to soil or dry conditions as *Klebsormidium*, CO_2_ could represent the preferred source of C_i_ for photosynthesis. However, to our knowledge, the mechanisms whereby these terrestrial streptophyte green algae attain C_i_ have not been investigated so far.

The aim of the present study is to perform a comparison of physiological traits in the genera *Klebsormidium* and *Zygnema* in relation to ecological parameters (light, water and C_i_) which characterize terrestrial habitats. For our experiments, we compared a new *Klebsormidium* isolate from an acidic environment with *K.* cf. *flaccidum* from a soil crust and a *Zygnema* sp. isolated from a sandy river shore. The latter two isolates have been characterized by means of phylogeny, structure and ultrastructure as well as some physiological aspects before (Karsten et al. 2013; Mikhailyuk et al. 2015; Herburger et al. 2015). These previous observations gave a solid basis for the present study; however, a direct comparison of these two genera in culture medium at the same culture age and with the same methods was not carried out before. We analysed their ability to employ photoprotective mechanisms and how they are linked to terrestrial light conditions. We also characterized the changes of the photosynthetic apparatus during a dehydration/rehydration cycle under different light regimes, aiming to define the role of dehydration in shaping species-specific photosynthesis. Finally, we assessed the presence of CCMs and tested if the acquisition of different C_i_ forms is related to the occurrence of these genera in different habitats.

## Materials and methods

### Species morphology and isolation

We used a *Zygnema* sp. (Culture collection of Algae Göttingen, SAG 2419, isolated from a sandy river shore, Herburger et al. 2015) and two *Klebsormidium* isolates. These included: (1) a new *Klebsormidium* isolate with long, tangled filaments, collected from the acidic (pH 4.3) environment of a former mining site termed Schwarzwand (47°9′ 36.84″N, 13° 13′13.28″E) (Großarl Valley in Salzburg, Austria, Adlassnig et al. 2013); and (2) the previously described *K. flaccidum* KUE1 (alpine biological soil crust, Tyrolean Alps, Austria) and grouping into the B-Clade according to ITS-phylogeny (Karsten et al. 2013). The latter species name was also modified into *K.* cf. *flaccidum* by Mikhailyuk et al. (2015) according to the observation that the filaments had a stronger tendency to disintegrate.

### Culture conditions

The two *Klebsormidium* isolates were grown in modified Bolds Basal Medium (MBBM) and *Zygnema* in standard BBM culture media, respectively, and buffered at pH 7.5 using 40 mmol L^−1^ Hepes. Cultures were incubated in a growth chamber with a temperature cycle of 20–15 °C of 16:8 h, and exposed to an incident photon flux density of 50–70 µmol photons m^−2^ s^−1^. All species were maintained in batch growth, using 200 mL Erlenmeyer flasks filled with a maximal culture volume of 100 mL. Cultures were refreshed with culture medium regularly (every 2 weeks) to maintain filaments concentration low and to avoid nutrients depletion in the medium. To test a possible/particular adaptation of *Klebsormidium* isolated from Schwarzwand to low pH environments, results from control experimental condition (pH 7.5) were compared to analyses performed on filaments transferred for 24 h into a MBBM pH 4.1 (buffered with 5 mM citric acid/Na-citrate, Gerloff-Elias et al. 2005). Prolonged (several days) exposure of filaments to low pH caused the culture medium to get ‘turbid’ which we considered unsuitable for further physiological experiments.

### Phylogenetic analyses

The new *Klebsormidium* strain from Schwarzwand was characterized by *rbc*L (large subunit of ribulose-1,5-bisphosphate carboxylase/oxygenase) marker, which is the most used molecular marker for this streptophytic green algae. The DNA from the strain was isolated according to the protocol of Ryšánek et al. (2015). The sequences of the *rbc*L gene were obtained using polymerase chain reaction (PCR) amplification with a Touchgene Gradient cycler (Techne, Cambridge, UK). The *rbc*L gene was amplified using the forward primer KF590 150 (5′-GAT GAA AAC GTA AAC TCT CAG C-3′) and the reverse primer *rbc*L-KR2 (5′-GGT TGC CTT CGC GAG CTA-3′) (Škaloud and Rindi 2013). Each 20 μL reaction solution for PCR was conducted as described by Ryšánek et al. (2015). The PCR protocol followed that of Škaloud and Rindi (2013). Sequencing reads were assembled and edited using SeqAssem software (Hepperle 2004). Newly obtained *Klebsormidium rbc*L sequence and the sequences available in the GenBank database were used to produce an alignment for phylogenetic analyses. The final alignment was constructed by ClustalW (Thompson et al. 1994) with MEGA v6.06 (Tamura et al. 2011). The aligned data set was analysed using Bayesian analysis (BI) with MrBayes v3.1.2 (Huelsenbeck and Ronquist 2001), maximum likelihood analysis (ML) with GARLI (Zwickl 2006), and maximum parsimony (MP) analysis with PAUP v4.0b10 (Swofford 2002). The evolutionary model used was the same as in Ryšánek et al. (2015). The BI analysis was performed using the priors set as default in MrBayes; the robustness of the tree topologies was assessed by bootstrapping the data set as described by Škaloud and Rindi (2013).

### Light- and transmission electron microscopy (TEM)

For light microscopy a Zeiss Axiovert 200 M microscope with a 100 × 1.3 NA objective lense was used and transmission electron microscopy was essentially carried out by a classical chemical fixation procedure as previously described (Holzinger et al. 2009). Transmission electron micrographs were captured with a TRS 2k SSCCD camera connected to a Zeiss Libra 120 TEM operated at 80 kV.

### Rapid light curves, NPQ and OJIP measurements

The light acclimation status and PSII properties of *Klebsormidium* and *Zygnema* were analyzed using a PAM 2500 fluorimeter (Heinz Walz, Effeltrich, Germany). Prior any measurements, samples were dark acclimated for 15 min. Rapid light curves (RLCs), as assessment of the photosynthetic response to rapid increase of light (every 30 s), were obtained by exposing cells to light intensities between 0 and 2014 µmol photons m^−2^ s^−1^. The RLCs were then fitted through the mathematical model of Walsby (1997). Fluorescence induction curves for NPQ estimation were obtained using 20 saturating light pulses (300 ms) upon cells exposed to an actinic light intensity of 1159 μmol photons m^−2^ s^−1^, and followed by a dark recovery time to monitor NPQ relaxation phase. The OJIP (O, origin; J and I, intermediated inflections; P, peak; Stirbet et al. 2014) transients were obtained by a multi turn-over flash generated using the default trigger pattern of PamWin-3 software (Poly300 ms.FTM).

### *P* vs *I* curves

Rates of photosynthetic O_2_ evolution as a function of irradiance (*P* vs *I* curve) were used as assessment of photosynthetic response towards relatively slower increase of light intensity. The *P* vs *I* curves were measured with a Presens Fibox 3 oxygen optode (Presens, Regensburg, Germany) fixed in a 3-mL thermostatic acrylic chamber (type DW1, Hansatech Instruments, Norfolk, UK) as in Kaplan et al. (2013). Prior each *P* vs *I* curve measurement, the cells were dark acclimated for 15 min with the final 5 min of this incubation period used to measure the dark respiration (*R*_d_). Following the dark period, *P* vs *I* curves were obtained by exposing the cell suspension to a progressive increase (every 5–10 min) of light intensities between 0 and 1520 μmol photons m^−2^ s^−1^. To avoid the possibility of photorespiration, during the experiments the O_2_ concentration in the chamber was maintained between 15 and 60% of air equilibrium (Pierangelini et al. 2014). The *P* vs *I* curves and photosynthetic parameters as maximum photosynthetic rate (*P*_max_), light harvesting (*α*), photoinhibition (*β*) and onset of light saturated photosynthesis (*I*_*k*_ = *P*_max_/*α*) were generated using the model of Walsby (1997). Results were normalized to Chl *a*. At the end of each *P* vs *I* curve, the algal suspension was filtered onto a Whatman GF/C glass microfiber filter, resuspended in 1 mL DMF (with overnight extraction), and the Chl *a* quantified photometrically using the equations of Porra et al. (1989).

### Dehydration and recovery experiment

To study the response of the photosynthetic apparatus when dehydration occurs at both sub- and saturating light intensity for photosynthesis, we performed a dehydration/rehydration experiment at two light regimes, 25 (LL, low light) and 185 (SL, saturating light) µmol photons m^−2^ s^−1^; measured with a Solar Light PMA 2132 cosine corrected PAR sensor connected to a Solar Light PMA 2100 radiometer (Solar Light Co., Inc., Philadelphia, PA, USA). Samples were placed under the same light source and the low light treatment was obtained using light screens. The dehydration and rehydration cycle were performed essentially as in Karsten et al. (2014, 2016) and in Herburger et al. (2015). Filaments of *Klebsormidium* and *Zygnema* were collected from the culture, re-suspend in 200 µL of fresh MBBM or BBM media and placed onto a 45-mm membrane filter (mixed cellulose ester, Whatman GmbH, Dassel, Germany). Filters were placed inside the desiccation chamber described in Karsten et al. (2014), and filled with 100 mL 3.5 mol L^−1^ KCl dehydrating solution. Dehydration was allowed to take place for 24 h. After this period, filaments on filters were rewetted with 200 µL of fresh culture medium and the KCl solution in the chamber replaced with 100 mL of tap water. The relative humidity (78.5–94%) during the experiment was recorded using a PCE-MSR145S-TH mini data logger (PCE Instruments, Meschede, Germany). For the assessment of the PSII proprieties, the PAM 2500 probe was placed outside the chamber at the fixed distance of 11 mm from the filter. The effective quantum yield $({\text{YII}} = (F_{m}^{\prime } - F)/F_{m}^{\prime } )$ was measured on filaments exposed to each respective light intensity. *F*_v_/*F*_m_ and OJIP transients were measured during the dehydration phase on dark acclimated filaments for 15 min. At the end of the rehydration phase of *Klebsormidium*, *F*_v_/*F*_m_ were measured on filaments collected from the filters and resuspended in fresh MBBM medium.

### pH drift and C_i_ acquisition

For the analysis of C_i_ acquisition in *Klebsormidium* and *Zygnema* we performed a pH-drift experiment as described by Maberly and Spence (1983) using an artificial assay medium (pH 7.5, alkalinity ~1 mEq L^−1^) prepared as in Lachmann et al. (2016). Filaments were harvested from the culture, washed in 20 mL of assay medium, placed in an air tight 25 mL glass vial (obtaining a Chl *a* concentration between 0.4 and 0.8 µg mL^−1^), and exposed to a maximal incident light intensity of 110 µmol photons m^−2^ s^−1^. The increase of pH of the assay medium was recorded every 30–60 min by quickly opening and introducing the pH probe into the vials. During the measures gas exchange was minimal since the pH probe fitted the vials aperture. At the end of pH drift incubation, the final alkalinity of assay medium was measured by Gran tritration and C_i_ speciation (CO_2_, HCO_3_^−^, CO_3_^−2^) calculated from the constants of Millero (1979) and the NBS pH scale, using the CO_2_Sys.xls application (Holland et al. 2012). The results were used to estimate the variation of the C_i_ forms during the course of the experiment, to calculate the maximal C_i_ uptake rate normalized to Chl *a* (extracted as described above), and calculate the quotient of final total C_i_ (*C*_T_) over final alkalinity (*C*_T_/Alk, Lachmann et al. 2016).

### Statistical analysis

Experiments were performed with at least three biological replicates. We tested the significance of mean differences among the three organisms using one-way ANOVA followed by Bonferroni’s multiple comparison test. The variation among means in relation to time was tested using two-way repeated-measures (RM) ANOVA. Comparison between two means was carried out by two-tailed *t* test. The analyses were performed using the software GraphPad Prism 5, setting the threshold of significance at 95%.

## Results

### Phylogenetic characterization, light and transmission electron microscopy of the new isolate

**Fig. 1:**
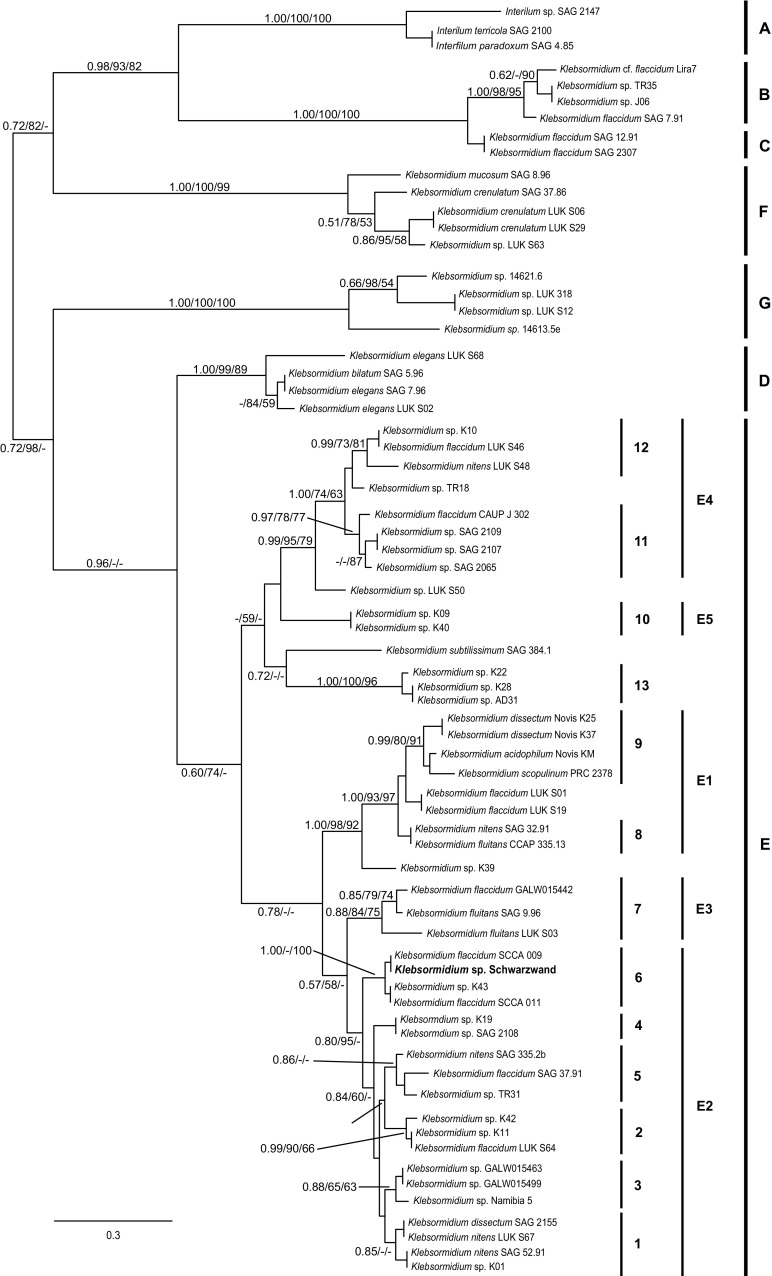
Phylogenetic tree obtained from Bayesian analysis based on *rbc*L dataset, showing the position of newly investigated strain of *Klebsormidium* sp. isolated from Schwarzwand and their relatives. Values at the *nodes* indicate statistical support estimated by MrBayes posterior probabilities (*left*), maximum likelihood bootstrap (*middle*), and maximum parsimony bootstrap (*right*). The clade numbering (*A*–*G*, *E1*–*E6*) follows Rindi et al. (2011) and clades (*1*–*13*) are according to Škaloud and Rindi (2013)

**Fig. 2:**
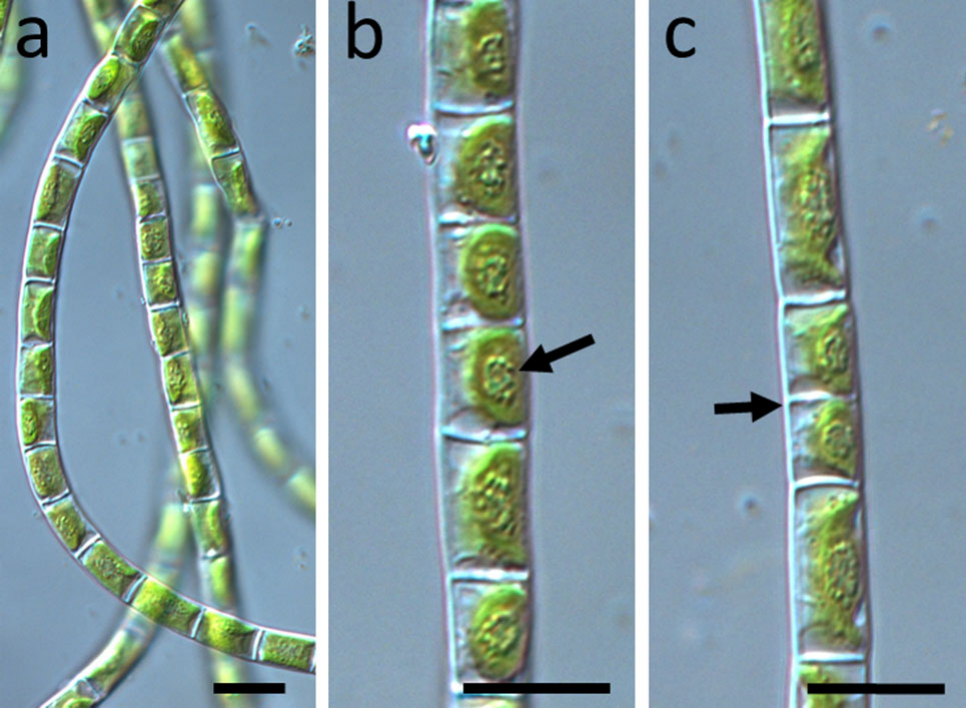
Light micrographs of *Klebsormidium* sp. (SCHW). **a** Overview of several filaments showing how they are among each other. **b** Detail of one filament showing the prominent pyrenoids (*arrow*) in the centre of the chloroplasts, **c** filament with cells that just divided (*arrow*), illustrating the different cell lengths. *Bars* 10 µm

**Fig. 3:**
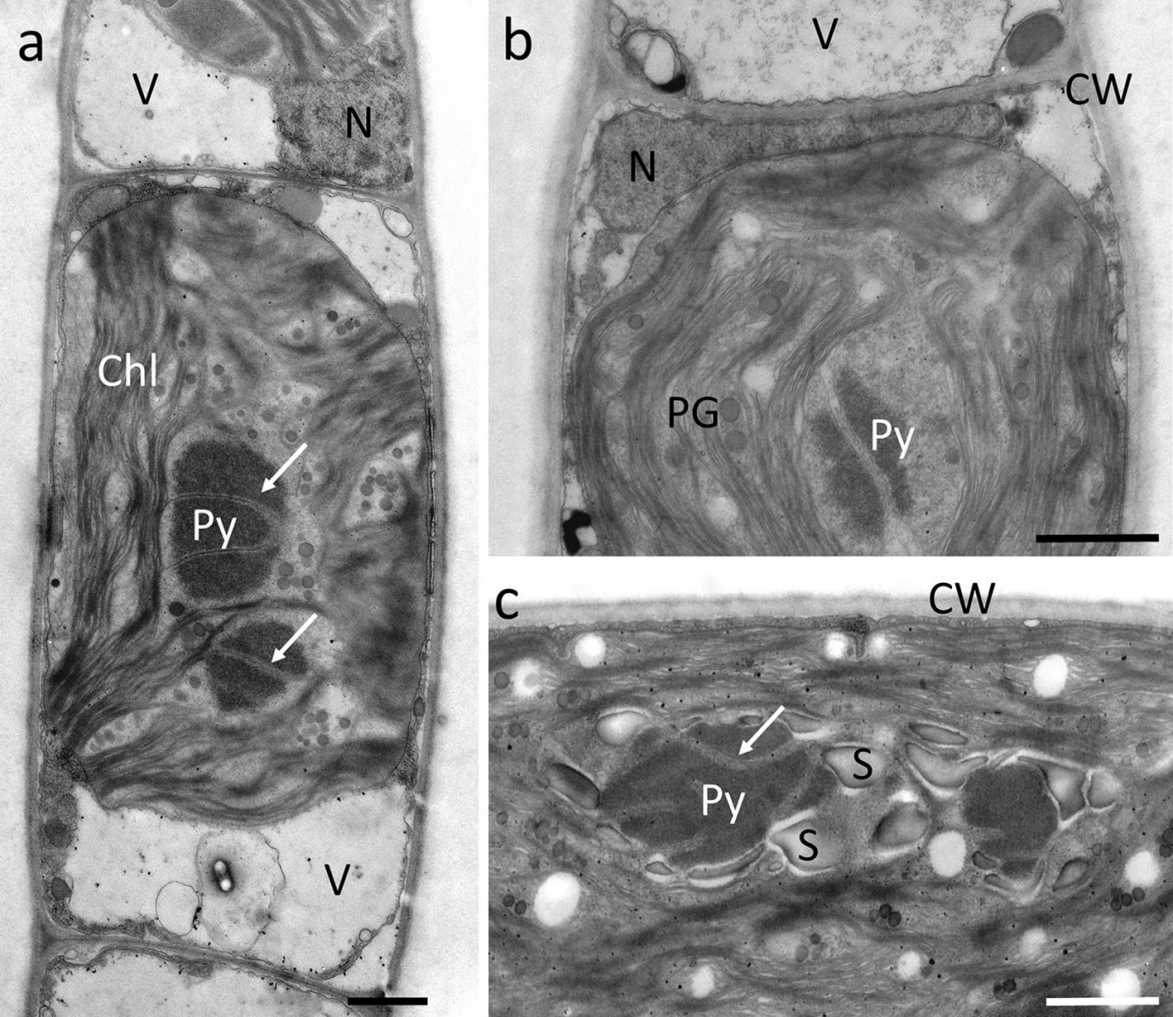
Transmission electron micrographs illustrating *Klebsormidium* sp. (SCHW). **a** Overview with parietal chloroplast that contains a central pyrenoid. Towards the edges of the cell, larger vacuoles are found, note the position of the nucleus in the upper cell. **b** Cell with elongated nucleus, positioned close to the cross wall, several plastoglobues can be found. **c** Detail of the chloroplast showing pyrenoids surrounded by starch grains and crossed by thylakoids. *Chl* chloroplast, *CW* cell wall, *N* nucleus, *PG* plastoglobules, *Py* pyrenoid, *S* starch grain. *Bars* 1 µm

While phylogenetic and morphological characterization of the *K.* cf. *flaccidum* strain KUE1 (Karsten et al. 2013; Mikhailyuk et al. 2015) and *Zygnema* sp. (SAG 2419, Herburger et al. 2015) were previously available, the new strain isolated from Schwarzwand, Austria, hereafter *Klebsormidium* sp. (SCHW), was found to group into clade E2 by *rbc*L analysis (Fig. 1). The cells had an average cell width of 5.5 (±0.5) µm and an average cell length of 9.3 (±1.8) µm, and a parietal chloroplast with a prominent pyrenoid surrounded by numerous starch grains (Fig. 2). The chloroplasts covered at least 2/3 of the inner cell surface. Transmission electron microscopy of the new isolate allowed to further characterize the subcellular organization. The chloroplasts contained prominent pyrenoids (Fig. 3) that were transversed by thylakoid membranes (Fig. 3a). Several pyrenoids were found to be surrounded by starch grains (Fig. 3). The chloroplasts contained several plastoglobules (Fig. 3a, b). The nucleus was found occasionally not in the typical central position, but close to the cross walls, sometimes drastically elongated (Fig. 3a, b).

### Maximum quantum yield and Non-photochemical quenching

**Fig. 4:**
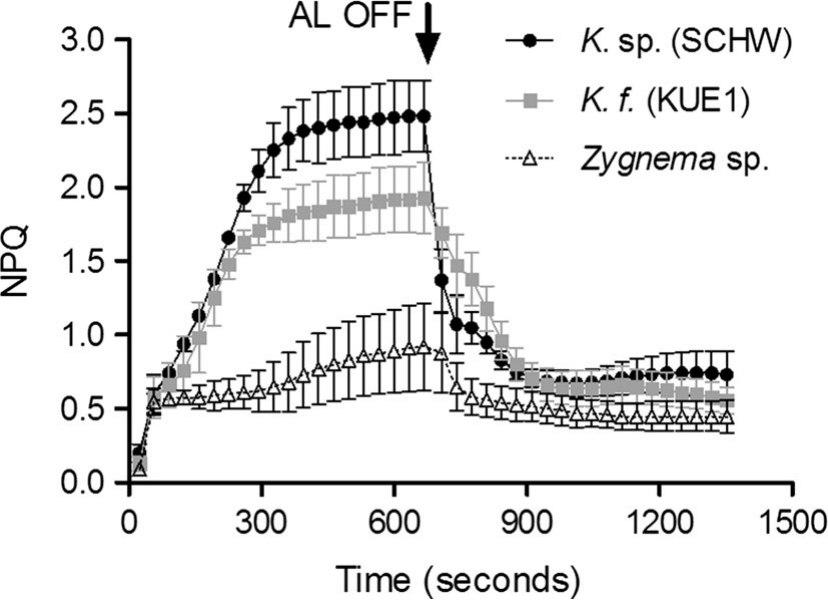
Kinetics of NPQ for *Klebsormidium* sp. (SCHW, *black circles*), *K.* cf. *flaccidum* (KUE1, *grey squares*) and *Zygnema* sp. (*white triangles*). *Arrow* indicates when the actinic light (AL) was turned off. Measures were performed from at least three independent replicates

Similar *F*_v_/*F*_m_ were found among *Klebsormidium* isolates and *Zygnema* (*P* = 0.1746, Table 1). The different NPQ kinetics between *Klebsormidium* and *Zygnema* are reported in Fig. 4. Compared to *Zygnema*, both *Klebsormidium* isolates showed a higher capacity to perform NPQ. Moreover, differently from *Zygnema*, the *Klebsormidium* NPQ was inducible and reaching full activation after a relative long time (~6 min) of exposure to strong actinic light. *Klebsormidium* sp. (SCHW) showed higher maximal NPQ (*P* = 0.0107) than *K.* cf. *flaccidum* (KUE1). The kinetics of the *Klebsormidium* NPQ induction reported here are comparable to the results of Gerotto and Morosinotto (2013).

**Table 1:** Maximum quantum yield, dark respiration and photosynthetic characteristics (*P* vs *I* curves) of the two *Klebsormidium* isolates and *Zygnema*

	*F*_v_/*F*_m_	*R*_d_^a^	*P*_max_^a^	*α* ^b^	*I*_*k*_^c^	*β* ^b^
*Klebsormidium* sp. (SCHW)	0.67 (0.04)	−29 (14)	197 (61)	7.28 (2.37)	30 (17)	−0.02 (0.02)
*K.* cf. *flaccidum* (KUE1)	0.70 (0.04)	−19 (29)	191 (59)	2.40 (0.70)*	79 (7)*	−0.00 (0.01)
*Zygnema* sp.	0.66 (0.05)	−38 (15)	208 (54)	3.15 (1.84)*	76 (22)*	−0.03 (0.02)

Values in brackets represent standard deviation (*n* ≥ 3) and asterisks indicate statistically significant differences from *Klebsormidium* sp. (SCHWs)

^a^µmol O_2_ mg Chl a^−1^ h^−1^

^b^µmol O_2_ mg Chl a^−1^ (µmol photons m^−2^ s^−1^)^−1^

^c^µmol photons m^−2^ s^−1^

### Slow vs rapid increase of light

**Fig. 5:**
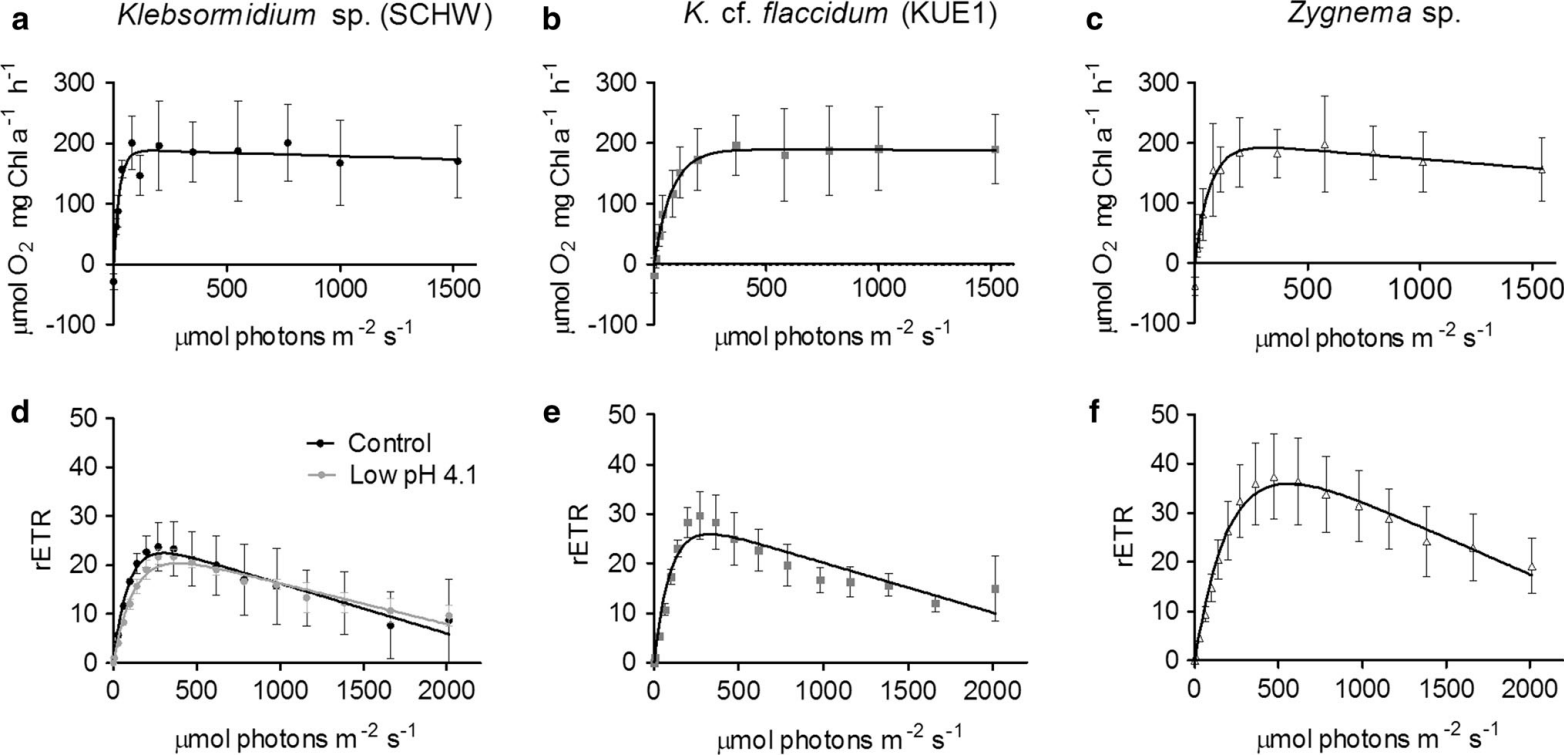
Photosynthetic characteristics of *Klebsormidium* and *Zygnema*. **a**–**c** *P* vs *I* curves measured as an assessment of the photosynthetic responses to slow light increase. **d**–**f** RLCs used as an assessment of the photosynthetic responses to rapid increase of light. **d** RLCs are included for *Klebsormidium* sp. (SCHW) exposed for 24 h at pH 4.1. *Vertical bars* indicate standard deviations of at least three independent replicates

To understand how the NPQ traits are linked to natural light conditions, we compared the results of *P* vs *I* curves with the RLCs. The results of *P* vs *I* curves, showing the photosynthetic responses of *Klebsormidium* isolates and *Zygnema* to relatively slow increase of light intensity, are reported in Fig. 5a–c and in Table 1. *R*_d_ (*P* = 0.3731) and *P*_max_ (*P* = 0.9023) were similar among the *Klebsormidium* isolates and *Zygnema.* The *α* was higher for *Klebsormidium* sp. (SCHW) than in *K.* cf. *flaccidum* (KUE1) and *Zygnema* (*P* = 0.0155). Reflecting the higher *α*, the *I*_*k*_ was found lower in *Klebsormidium* sp. (SCHW) (*P* = 0.0137). *Klebsormidium* sp. (SCHW) showed negligible *β*, whereas for *K.* cf. *flaccidum* (KUE1) *β* was null. On the other hand, although statistically weak (*P* = 0.0574), *Zygnema* showed higher tendency to photoinhibition. Figure 5d–f show the responses of *Klebsormidium* isolates and *Zygnema* to a comparatively faster increase of light intensity (RLCs curves). The most striking observation is that with RLCs all species studied had higher susceptibility to high light intensity (*β*) than during the *P* vs *I* curves.

### Low pH experiment

Short-term exposure (24 h) of *Klebsormidium* sp. (SCHW) to pH 4.1 caused a decline of the *F*_v_/*F*_m_ from 0.67 (±0.02) down to 0.51 (±0.03) (*P* = 0.0019), suggesting the occurrence of changes/damages at the PSII. The RLCs (Fig. 5d) highlighted a decline of *α* (*P* = 0.0134) but rETR_max_ was not altered under the low pH condition (*P* = 0.5671).

### Dehydration and rehydration

**Fig. 6:**
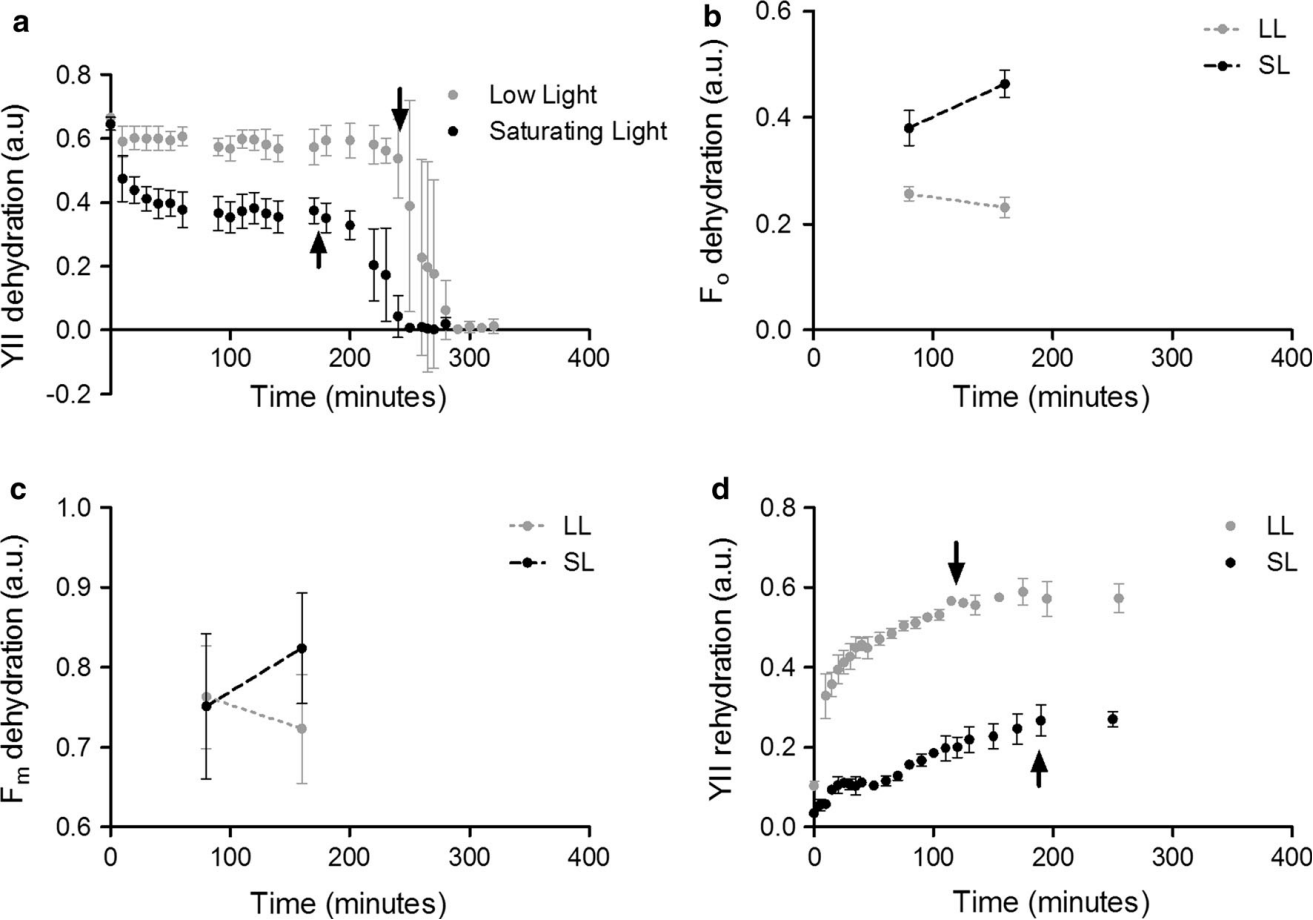
Variations of fluorescence parameters for *Klebsormidium* sp. (SCHW) during dehydration-rehydration experiment under low light (LL, 25 μmol photons m^−2^ s^−1^) and saturating light for photosynthesis (SL, 185 μmol photons m^−2^ s^−1^). **a** YII, effective quantum yield during dehydration, with *arrows* pointing the time differences for the onset of YII decline. **b** *F*_o_, minimal fluorescence during dehydration. **c** *F*_m_, maximal fluorescence during dehydration. **d** Recovery of YII during rehydration (after 24 h of being in a dehydrated state), with *arrows* indicating the time when YII reaches stable values. *Vertical bars* indicate standard deviations of three replicates

**Fig. 7:**
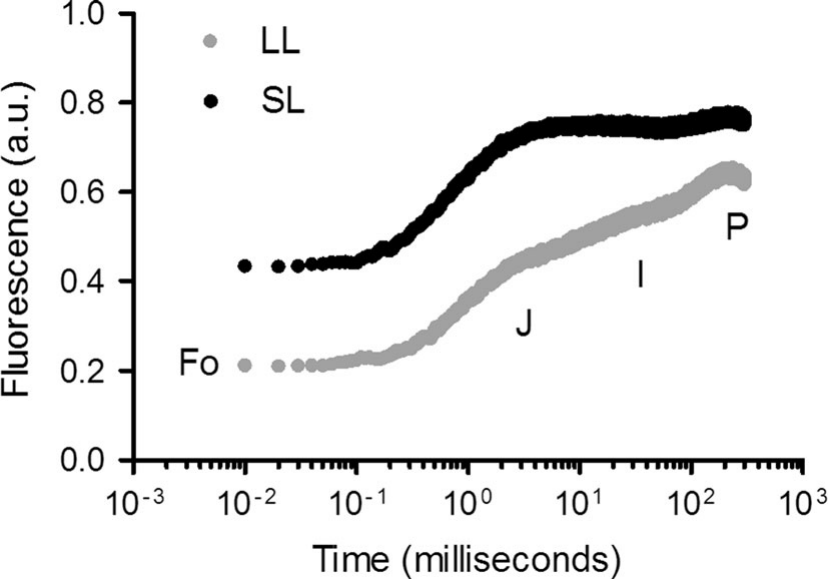
O-J-I-P transients analysed on *Klebsormidium* sp. (SCHW) during dehydration under low light (LL, 25 μmol photons m^−2^ s^−1^) and saturating light for photosynthesis (SL, 185 μmol photons m^−2^ s^−1^). Results are the average of measurements performed on three independent replicates

As reported by Herburger and Holzinger (2015) *Zygnema* was more sensitive to dehydration than *Klebsormidium*. The YII rapidly declined during dehydration and did no recover after 24 h (Fig. S1). Contrary, *Klebsormidium* sp. (SCHW) tolerated longer dehydration periods and quickly recovered even after 24 h of being in a dehydrated state (Fig. 6). Due to the dependence of the YII on the light acclimated state (which induces non-photochemical down-regulation and reaction centres closure), the absolute differences in YII between the LL and SL treatments can be attributed to the different light regime at which the cells were exposed during the fluorescence measures. Yet, our results showed that the capacity of *Klebsormidium* to tolerate dehydration and rehydration cycle was influenced by the light exposure. During dehydration, the YII (Fig. 6a) of cells exposed to SL started to drop down to null values earlier (40 min) than for cells under the LL treatment. In SL, the *F*_o_ measured on dark acclimated cells was higher than in cells under LL (two-way ANOVA RM, *P* = 0.0002; Fig. 6b) and showing an increasing trend. Since no changes were observed in the *F*_m_ (two-way ANOVA RM, *P* = 0.4683; Fig. 6c), the decrease of *F*_o_ is considered the cause for the *F*_v_/*F*_m_ decline (two-way ANOVA RM, *P* = 0.0002; data not shown). The OJIP transients measured on the same dark acclimated cells confirmed these results, showing a higher *F*_o_ (*P* = 0.0109), unchanged *F*_m_ (*P* = 0.2043) and lower *F*_v_/*F*_m_ (*P* = 0.0029) in SL than in LL cells (Fig. 7; Table 2). As well as for dehydration, exposure of cells to SL influenced the recovery of the photosynthetic machinery during rehydration phase (Fig. 6d). While for the cells at LL the YII rapidly returned (within 2 h) to values as high as those measured at dehydration, the YII recovery for cells in SL was slower and stopped at ~73% of the YII values measured during dehydration (Fig. 6a). Consistently, after 4 h in rehydrated condition, cells in the SL showed lower *F*_v_/*F*_m_ (0.47 ± 0.03) than at LL which was fully recovered (0.67 ± 0.02) (*P* = 0.0011), indicating the presence of PSII damages which impaired the cells to recover their photosynthetic capacity.

**Table 2:** Parameters extrapolated from the O-J-I-P transients during *Klebsormidium* sp. (SCHW) dehydration (±standard deviation) under low light (25 μmol photons m^−2^ s^−1^) and saturating light for photosynthesis (185 μmol photons m^−2^ s^−1^)

OJIP parameters during dehydration		Low light	Saturating light
*F*_o_	Minimal fluorescence in dark	0.22 (0.06)	0.44 (0.06)*
*F*_m_	Maximal fluorescence in dark	0.63 (0.14)	0.76 (0.07)
*F*_v_/*F*_m_	Maximum quantum yield	0.66 (0.06)	0.43 (0.03)*
*V*_j_	Fluorescence at the J-step	0.48 (0.17)	0.83 (0.07)*

The parameter *V*_j_ was calculated according to the equation of Strasser et al. (2000). Asterisks indicate statistically significant differences from the low light treatment

### pH-drift and C_i_ acquisition

**Fig. 8:**
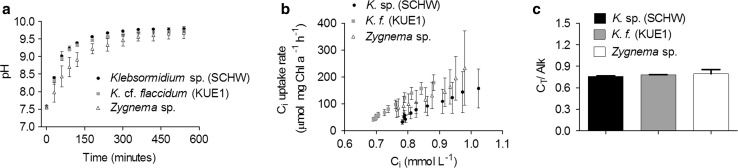
**a** Result of pH–drift experiment carried out on *Klebsormidium* sp. (SCHW), *K.* cf. *flaccidum* (KUE1) and *Zygnema*. **b** Variation of C_i_ uptake as a function of C_i_ concentration in the assay medium. **c** *C*_T_/Alk quotients. *Vertical bars* indicate the standard deviation calculated on at least three independent measures

The rise of pH during the pH-drift experiment is shown in Fig. 8a. The pH increased rapidly from 7.6 to ~9.0 but the following increase up to final and stable values of ~9.7 occurred more slowly. The rates of C_i_ uptake as a function of C_i_ (Fig. 8b) did not show any species-specific variation. Similarly, the *C*_T_/Alk quotients (Fig. 8c) were not statistically different between *Klebsormidium* isolates and *Zygnema* (*P* = 0.5279).

## Discussion

In this work, we investigated the eco-physiological traits that make *Klebsormidium* and *Zygnema* capable to successfully colonize terrestrial habitats. We showed that photosynthetic characteristics are distinct between *Klebsormidium* and *Zygnema* (NPQ, photoinhibition), reflecting their preference for different light regimes in natural ecosystems. These streptophyte green algae possess comparable C_i_ acquisition traits, indicating no genera-specific adaptation to habitats but rather an overall C_i_ acquisition adaptation to terrestrial life. The sensitivity of *Klebsormidium* to light conditions during cellular water loss, emphasises the importance of considering multiple environmental factors when studying the effectiveness of mechanisms involved in protection of the photosynthetic apparatus during dehydration.

Regarding the newly isolated *Klebsormidium* strain from an acidic environment (Schwarzwand, Austria), the physiological comparison showed that rates of *R*_d_ and *P*_max_ were similar to *K.* cf. *flaccidum* (KUE1). This is interesting, as these two *Klebsormidium* strains belong to rather distinct clades according to molecular phylogeny. While by means of *rbc*L phylogeny *Klebsormidium* sp. (SCHW) was grouping into clade E2, according to the terminology of Rindi et al. (2011), the *K.* cf. *flaccidum* strain previously isolated grouped into clade B (Karsten et al. 2013) or the combined subclades B/C (Mikhailyuk et al. 2015). However, the higher *α* in *Klebsormidium* sp. (SCHW) suggests a relatively higher ability to tolerate lower light and this could be attributed to the morphological differences between the two strains. In fact, the longer and tangled filaments of *Klebsormidium* sp. (SCHW) in comparison to *K.* cf. *flaccidum* (KUE1) are expected to make cells of this isolate to occur in a comparatively more self-shaded environment. The *Klebsormidium* sp. (SCHW) also showed negative effects on *F*_v_/*F*_m_ by low culture pH, excluding the possibility that this could be a different ecotype. We, therefore, suggest that *Klebsormidium* occurrence in ecological niches with stressful physiological conditions (i.e., low pH, potentially elevated heavy metal concentration) is favoured by the reduced competition with other species. Although there are indications that some *Klebsormidium* isolates showed preferences for certain substrata with differing pH (Ryšánek et al. 2016).

The new *Klebsormidium* sp. (SCHW) isolate was also characterized by light- and transmission electron microscopy, to get further insights into the subcellular organization. Prominent pyrenoids, surrounded by numerous starch grains are observed. Like for *Chlamydomonas*, the thylakoid membrane traversing the pyrenoid could be involved in CCM activity by containing a lumenal carbonic anhydrase which aids the conversion of HCO_3_^−^ (transported inside the lumen from the stroma) into CO_2_ and deliver CO_2_ in proximity to Rubisco (Moroney et al. 2011; Meyer and Griffiths 2013).

The occurrence of numerous plastoglobules in chloroplasts is usually an indication for thylakoid membrane degradation (e.g. Holzinger et al. 2011). One interesting observation was that the nuclei were frequently found in a position close to the cross cell walls. This is usually only the case after cell division (e.g., Lokhorst and Star 1985) and the nucleus moves then back to its central position. The frequent occurrence of this position either could point towards increased division activity or could be an indication of incomplete cell divisions.

For both *Klebsormidium* isolates and *Zygnema* the *I*_*k*_ parameter was found to be low and very similar, suggesting a low light adaptation for these organisms (Herburger et al. 2015; Karsten et al. 2016). The presence of such low light adaptation contradicts the intuition that algae of the soil crusts (including those from high Alpine environments) may experience direct and intense solar radiation in natural conditions (Gray et al. 2007; Karsten et al. 2010). In contrast, the chlorophyte *Chlorella ohadii* isolated from highly irradiated desert soil crust can tolerate light intensities as high as 3500 µmol photons m^−2^ s^−1^ (Treves et al. 2013, 2016). It has been suggested that low light adapted terrestrial species occur in micro-environments of the soil crust where they are protected from incident light, or might be relived from stressful light conditions by filaments self-shading (Gray et al. 2007; Karsten et al. 2016). A similar field observation was made in a desert cyanobacterial soil crust, where maximal photosynthesis occurred beneath the surface where cells are sheltered (Raanan et al. 2016b).

Despite the similar photosynthetic light characteristics between *Klebsormidium* and *Zygnema*, drastic differences were observed in their photoprotective mechanisms. Gerotto and Morosinotto (2013) described that for *Klebsormidium* and *Zygnema* the major component of NPQ is represented by the energy-dependent qE, which is regulated by lumen acidification (Roach and Krieger-Liszkay 2014). For *Klebsormidium* the maximal NPQ was higher than in *Zygnema*. Moreover, for this genus the NPQ was inducible and its full capacity was reached relatively slowly during exposure to strong light, particularly in comparison to other streptophyte algae (Gerotto and Morosinotto 2013) or some aquatic microalgae (Kotabová et al. 2011; La Rocca et al. 2015). Lumen acidification and NPQ induction may also involve the presence of an active cyclic electron flow around PSI (CEF-PSI) (Golding and Johnson 2003; Joliot and Johnson 2011), and whose activity has been shown in *K. flaccidum* (Hori et al. 2014). Contrary to *Klebsormidium*, *Zygnema* showed a lower and a more constitutive capacity to perform NPQ. Interestingly, *Zygnema* differs from *Klebsormidium* also for not having LHCSR involved NPQ activation but PSBS protein, resembling the NPQ activation in vascular plants (Gerotto and Morosinotto 2013). However, the presence of PSBS may not be necessarily related to the closer phylogenetic position of *Zygnema* to these organisms (Christa et al. 2017).

The differences in NPQ capacity and kinetics are linked the different responses of *Klebsormidium* and *Zygnema* photosynthetic apparatus to slow or rapid increase of light intensity. When the light in the environment increased relatively slow (as during *P* vs *I* curves), the high NPQ capacity prevented the low light adapted photosynthetic apparatus of *Klebsormidium* from being photoinhibited, even at light intensities as high as 1500 μmol photons m^−2^ s^−1^. However, due to the slow NPQ activation, the photosynthetic apparatus was prone to suffer from photoinhibition if the light in the environment increases rapidly (RLC curves). During the RLCs, the activation of NPQ under strong light may also explain the inflection of the slope of photoinhibition, particularly noticeable for *K.* cf. *flaccidum* (KUE1). From the eco-physiological point of view, these photosynthetic responses reflect the *Klebsormidium* adaptation to highly irradiated terrestrial environments where increases or changes of light intensity (related to solar position in the sky or cloud cover) occur slowly during the day. For *Zygnema* with a much lower and not inducible NPQ capacity, the presence of photoinhibition was found under both slow and rapid increase of light. This suggests that *Zygnema* could prefer environments with more shaded conditions than *Klebsormidium*. In addition to NPQ, presence of different UV protecting compounds as phenolics in *Zygnema* (Pichrtová et al. 2013) or mycosporine-like amino acids in *Klebsormidium* (Kitzing et al. 2014) may further modulate the resistance under natural light conditions.

For species of the soil crust, slow increases of light intensity during mornings can also be associated with dehydration (Raanan et al. 2016a). For the young *Zygnema* culture investigated in the present study, we found no resistance to desiccation as previously described (Herburger et al. 2015). For *Zygnema* another strategy might be very important, the ability to form pre-akinetes that accumulate lipids (Pichrtová et al. 2016), and showed a reduced physiological activity, beneficial to tolerated desiccation stress (e.g., Herburger et al. 2015; Pichrtová et al. 2014). However, these pre-akinetes were not subject of the present study. In the case of the desiccation tolerant *Klebsormidium*, exposure to relatively high light intensity (our SL condition) during dehydration compromised the photosynthetic apparatus functioning, similarly to other terrestrial microalgae (Gray et al. 2007). Under dehydration in SL, the most noticeable change in PSII fluorescence signal was the increasing minimal *F*_o_. This result is analogous to what has been previously observed for the marine green macroalgae *Ulva*, where the *F*_o_ increased during, at least for the first part, the dehydration experiment (Gao et al. 2011). The authors suggested that the *F*_o_ increase is associated to a reversible inactivation of PSII reaction centres or the separation of the antenna complex from the PSII. However, these alterations seem not to happen at PSII level in *Klebsormidium* since the *F*_m_ values were not affected during dehydration. It is also known that *F*_o_ is influenced by the dark reduction of the plastoquinone (PQ) pool (Groom et al. 1993; Stirbet et al. 2014). Complementary to higher *F*_o_, the OJIP analysis revealed an increase in the J step (*V*_j_) with values as high as the P step (*F*_m_), and such changes have been associated to a higher reduction PQ pool (Tóth et al. 2007). We, therefore, relate the increasing *F*_o_ during dehydration in SL to an electron accumulation and a reduced state of the plastoquinone (PQ) pool along the electron transport chain (ETC). The progressive accumulation of electrons in the ETC under this condition, may eventually enhance radiative charge recombination events, leading to singlet oxygen production (Ohad et al. 2010, 2011) and thus, being responsible for the hastened PSII inactivation (YII decline) and impaired recovery after rehydration. It must also be pointed out that the SL used for this experiment is rather low (185 μmol photons m^−2^ s^−1^) compared to full sunlight intensity of an Alpine ecosystem. We also showed that under culture (i.e., hydrated) conditions *Klebsormidium* can tolerate up to eightfold this level of light intensity. We suggest that the reduction of the PQ (with the consequential damages on PSII) caused by relatively high light during dehydration events is an important physiological driver which shapes the species adaptation and occurrence towards low light.

The (over) reduction of the ETC during dehydration in the SL could be linked to different physiological alterations. Holzinger et al. (2014) measured a decline in *Klebsormidium* CO_2_ consumption rate during the dehydration phase. Thus, it is highly possible that electrons could be accumulated along the ETC following an imbalance between the excitation arriving at PSII, and the ability to remove electrons from the ETC, using them for CO_2_ fixation (Shimakawa et al. 2017). In conjunction, alterations of mechanisms involved in the redox regulation of the PQ may also take place (Rumeau et al. 2007). It has been proposed that exposure to water loss and/or high light promotes the activity of CEF-PSI over the linear electron flow, aiding lumen acidification and inducing NPQ for PSII protection (Golding and Johnson 2003; Miyake et al. 2005; Gao et al. 2011; Meneghesso et al. 2016). Taking into consideration that a CEF-PSI (mediated through the NAD(P)H dehydrogenase complex) is possibly operative in *Klebsormidium* (Hori et al. 2014), it is reasonable to hypothesise that even for our *Klebsormidium* strain the activity CEF-PSI could be intensified under dehydration at SL, and thus contributing to PQ pool reduction.

Although *Klebsormidium* and *Zygnema* have distinguished light photosynthetic characteristics, we show that their photosynthetic C_i_ acquisition is identical. The results of the pH-drift experiment reflected the cells ability to extract different C_i_ forms from the water environment (Maberly and Spence 1983). The pH increase up to final values of ~9.7 suggests that *Klebsormidium* and *Zygnema* are able to uptake both CO_2_ and HCO_3_^−^ for photosynthesis, and thus providing further evidence that these organisms have functional CCMs (Maberly et al. 2009). The high *C*_T_/Alk quotients are similar to other CO_2_-users, as *Chlamydomonas* sp. and planktonic desmids (Zygnematophyceae; Spijkerman et al. 2005; Lachmann et al. 2016). This, however, could be an indication that both *Klebsormidium* and *Zygnema* have a preference for CO_2_. This would be particularly relevant for *Klebsormidium* whose occurrence is restricted to soil and aero-terrestrial environments, where HCO_3_^−^ is not available for photosynthesis. These results do not support our hypothesis that *Klebsormidium* and *Zygnema* with different restriction to water (or isolated from acidic environment as in Lachmann et al. 2016), might possess distinguished C_i_ acquisition modes. Rather, they lead to the conclusion that these organisms acquired similar adaptations of their C_i_ acquisition during land colonization. Further studies are necessary to fully describe the terrestrial adaptation of their CCMs. Terrestrial streptophyte species are expected to be sensitive to spatial and temporal variation of CO_2_ in the environment. Spatially, CO_2_ variations could be linked to soil respiration which, stimulated by flux of organic matter, represents an input of CO_2_ (Suseela et al. 2012; Ng et al. 2014; Raven and Colmer 2016). Under the global change scenario, the long-term increase of atmospheric pCO_2_, predicted to reach values as high as 1000 ppmv by the end of twenty-first century (IPCC 2014), could cause genotypic changes (Collins and Bell 2004).

In conclusion, our work demonstrated that *Klebsormidium* and *Zygnema* possess distinguished photosynthetic traits which allow them to occur under different light regimes. These physiological traits might be the consequence of several adaptations acquired during their land colonization. Terrestrial environmental conditions as high irradiation and desiccation may have been counteracting forces shaping their photosynthetic apparatus. The high light may have favoured the acquisition of photoprotective mechanisms which allow them to occur in elevated light regimes (Alboresi et al. 2008, 2010). Opposite, the alterations of the ETC (leading to PSII damages) during dehydration under illuminated conditions may have favoured a shade adaptation. This is reflected by organisms such as *Klebsormidium* with a low light adapted photosynthetic apparatus but tolerant to high light intensity. It is also interesting that *Klebsormidium* and *Zygnema*, from different locations, habitat preferences and evolutionary positions share a similar C_i_ acquisition mode. It is known that atmospheric variations of CO_2_/O_2_ through geological time have given origin to diverse CCMs in aquatic algae (Raven et al. 2012, 2017; Hagemann et al. 2016). Genetic and molecular characterizations of CCMs in streptophyte algae are currently missing in the literature, although these could provide a useful insight on how atmospheric CO_2_ conditions have influenced land colonization by photosynthetic organisms.

### *Author contribution statement*

MP and AH designed the research and wrote the manuscript. MP conducted the physiological experiments. AH performed the light- and transmission electron microscopy. DR carried out the phylogenetic analysis. IL and WA collected and provided *Klebsormidium* sp. (Schwarzwand, Austria). All authors read and approved the manuscript.

## Electronic supplementary material

Below is the link to the electronic supplementary material.

Supplementary material 1 (TIFF 930 kb)

## Abbreviations

CEF-PSI: Cyclic electron flow around PSI
C_i_: Inorganic carbon
CCMs: CO_2_-concentrating mechanisms
ETC: Electron transport chain
LL: Low light
NPQ: Non-photochemical quenching
PQ: Plastoquinone
RLCs: Rapid light curves
SL: Saturating light
