# Supplementary figures and images for: Terrestrial adaptation of green algae Klebsormidium and Zygnema (Charophyta) involves diversity in photosynthetic traits but not in CO2 acquisition
Source: Planta. 2017 Jul 18;246(5):971–86. doi: 10.1007/s00425-017-2741-5 (PMC5633629; doi:10.1007/s00425-017-2741-5)

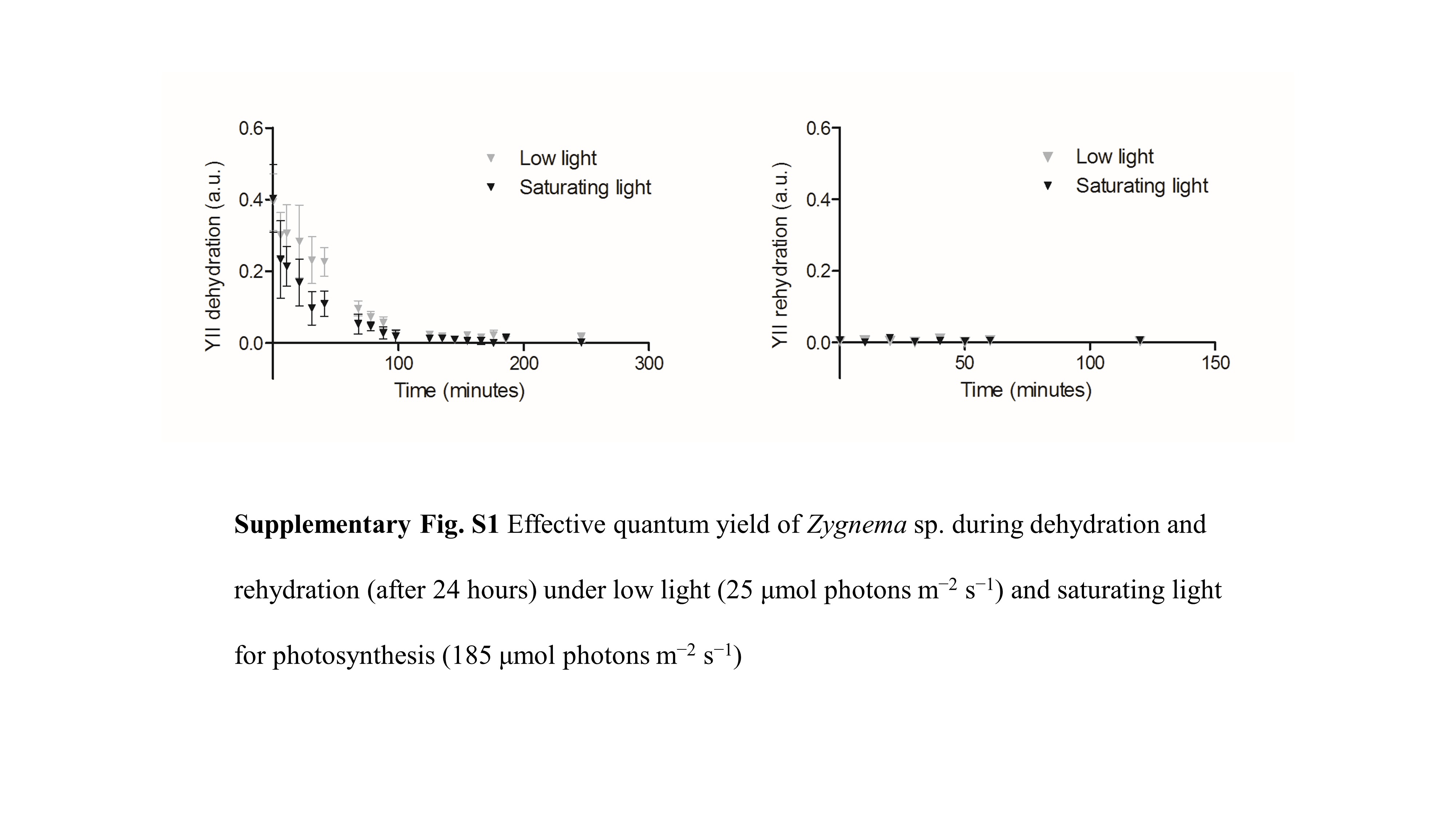

Supplement: Supplementary file 1 — Supplementary material 1 (TIFF 930 kb) [file 425_2017_2741_MOESM1_ESM.tif]
